# Down-regulated resistin level in consequence of decreased neutrophil counts in untreated Grave's disease

**DOI:** 10.18632/oncotarget.12019

**Published:** 2016-09-14

**Authors:** Ying Peng, Yicheng Qi, Fengjiao Huang, Xinxin Chen, Yulin Zhou, Lei Ye, Weiqing Wang, Guang Ning, Shu Wang

**Affiliations:** ^1^ Shanghai Clinical Center for Endocrine and Metabolic Diseases, Department of Endocrinology and Metabolism, Ruijin Hospital, Affiliated to Shanghai Jiao-Tong University School of Medicine, Shanghai 200025, China; ^2^ Laboratory of Endocrinology and Metabolism, Institute of Health Sciences, Shanghai Institutes for Biological Sciences (SIBS), Chinese Academy of Sciences (CAS) & Shanghai Jiao Tong University School of Medicine (SJTUSM), Shanghai 200025, China

**Keywords:** Graves' disease, resistin, neutrophils, T3

## Abstract

Resistin, belongs to cysteine-rich secretory protein, is mainly produced by circulating leukocytes, such as neutrophils monocytes and macrophages in humans. To date, few but controversial studies have reported about resistin concentrations in hyperthyroid patients, especially in Graves' disease (GD). We undertaked a controlled, prospective study to explore the serum resistin concentration in GD patients before and after -MMI treatment. In addition, we also investigated the main influencing factor on serum resistin level and discuessed the potential role of serum resistin plays in GD patients. 39 untreated GD (uGD) patients, including 8 males and 31 females, were enrolled in our investigation. All of these patients were prescribed with MMI treatment, in addition to 25 healthy controls. Anthropometric parameters and hormone assessment were measured. Enzyme-linked immunosorbent assay was used to detect serum resistin concentration in different stages of GD patients. Furthermore, neutrophil cell line NB4 with or without T3 treatment to detect the effect of thyroid hormones on resistin expression. The serum resistin level and neutrophil counts in untreated GD patients were significantly declined. And all of these parameters were recovered to normal after MMI treatment in ethyroid GD (eGD) and TRAb-negative conversion (nGD) patients. Resistin concentration exhibited a negative correlation with FT3 and FT4, but a positive correlation with absolute number of neutrophiles in uGD patients, whereas did not correlate with thyroid autoimmune antibodies and BMI. Neutrophile cell line, NB4, produced decreased expression of resistin when stimulated with T3. Our study showed a decrease of serum resistin level in GD patients and we suggested that the serum resistin might primarily secreted from circulating neutrophils and down-regulated by excessive thyroid hormones in GD patients.

## INTRODUCTION

Resistin, named by Steppan et al. in virtue of its crucial role in insulin resistance (resist to insulin) in mice, is a serine/cysterin-rich secretory protein [1, 2]. In rodents, it is primarily produced in adipocytes and influenced by genetic and diet, causing increased release of resistin in mouse models of obesity [3]. It has been demonstrated that mouse resistin could impair insulin sensitivity by increasing hepatic gluconeogenesis [4, 5]. In addition, mouse resistin has been shown to inhibit insulin-stimulated glucose uptake in skeletal muscle [6, 7] and adipocyte cells [1] itself. What astonished us was that the robust effect of resistin on insulin resistance in rodent was not successfully reproduced in human and the interaction of human resistin and obesity also showed controversial results [8]. First and foremost, human resistin is primarily secreted from inflammatory cells, such as monocytes and neutrophils, whereas micro-concentration in human adipocytes [9, 10]. Though associated with obesity and diabetes in population studies, resistin is thought to be secreted from infiltrated macrophages rather than adipose tissue itself. Resistin mediates the recruitment of immune cells by stimulating pro-inflammatory factors, leading to a chronic low-grade sub-clinical inflammation zone accompanying metabolism disorders [11–13].

Graves' disease (GD) acts as the most common cause of hyperthyroidism and a typical tissue-specific autoimmune endocrine disease with abnormally infiltrated B and T lymphocytes [14]. Abnormalities in local and circulating inflammatory factors and chemokines have been documented in contributing the development of GD [15, 16]. Meanwhile, hyperthyroidism is often associated with metabolism disorders. Gluconeogenesis is increased and glycogen synthesis is decreased in subclinical and overt hyperthyroidism, as compared to euthyroidism [17, 18]. In adults, increasing levels of TSH have been shown to be associated with increased total cholesterol [19, 20], LDL cholesterol [20, 21], non-high-density lipoprotein (HDL) cholesterol, triglycerides [20] and with decreased HDL cholesterol [20, 21]. Besides, adipokine such as adiponectin, leptin and visfatin could be affected by thyroid function [21–23]. Thus, as both adipokine and inflammatory factor, human resistin is deserved to be investigated about the role it played in immune dysfunction and metabolism disorder of GD.

The first such report found that patients with hyperthyroidism had less serum resistin level than euthyroidism controls and its concentration was not modified after attainment of euthyroidism. But after adjusting for BMI, the serum resistin exhibited a remarkable reduction. So authors suggested that resistin might be involved in the insulin resistance state that connected with thyrotoxicosis [24]. Similar to the result above, Bossowski A *et al*. also found a reduction of serum resistin in GD patients compared with simple goiter and Hashimoto's thyroiditis patients [25]. There were also several studies demonstrating that resistin is up-regulated in hyperthyroidism patients and returned to normal range after normalizing thyroid hormones [26, 27]. In addition, there was an investigation exhibited no change in serum resistin level in hyperthyroid patients compared with euthyroid healthy participants [28].

Concerning limited exploration of serum resistin levels in Chinese GD population and the controversial conclusion obtained so far, we want to investigate the serum resistin levels in GD patients before and after recovering the thyroid function. Meanwhile, we also explore the main source of serum resistin and effectors invloved in its expression.

## RESULTS

### Serum resistin levels in GD patients

**Figure 1:**
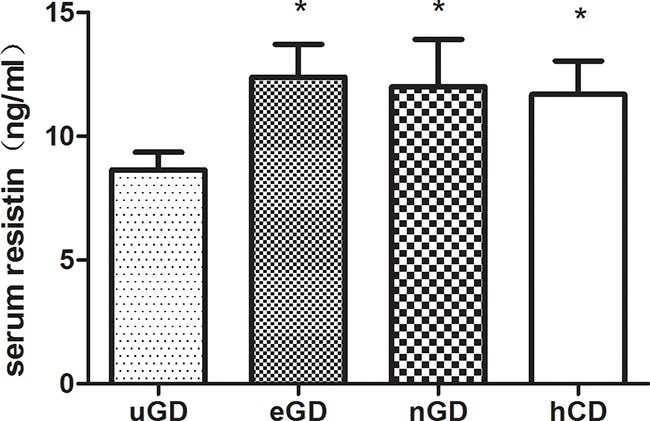
Serum resistin concentrations in GD patients and healthy controls Serum resistin level in untreated GD patients (n=39) decreased compared with and healthy controls(m=25). The resistin level in uGD patients increased to normal after normalization of thyroid hormones (eGD) and TRAb negative-conversion. Serum resistin was measured by enzyme-linked immunosorbent assay (ELISA). All data were presented as mean ± SEM. * : compared with uGD.* P<0.05.

To detect the serum resistin levels in GD patients, we enrolled 25 healthy volunteers and 39 untreated GD (uGD) patients. All uGD patients had increased concentrations of FT3 and FT4 and suppressed levels of s-TSH, whereas after 3-4 months of treatment with anti-thyroid drug MMI, the concentrations of thyroid hormones were recovered to normal, but TRAb level was still relatively high in eGD. And after 1-3 years of treatments, all the patients obtain normal concentrations of thyroid hormones and TRAb levels, which belong to nGD (Table 1). Patients with hyperthyroidism showed a decrease in serum resistin level compared with healthy control subjects (8.63 ± 4.41 vs. 11.69 ± 6.44 pg/ml; p=0.03). After adjustment for BMI, resistin levels maintained lower than healthy controls. Meanwhile, the resistin level was increased to normal range both in euthyroid GD (12.39±8.28 ng/ml) and TRAb negative-conversion patients (12.55±12.03 ng/ml) after effective MMI treatments (Figure 1). Furthermore, the correlation coefficients between serum resistin levels and the typical clinical parameters of GD were calculated. Interestingly, the results presented that resistin level was negatively correlated with FT3 (r=−0.375; p=0.022) and FT4 (r=−0.352; p=0.028), but not with TSH (r=−0.156; p=0.342), TRAb (r=−0.199; p=0.224), TPOAb (r=0.57; p=0.735), and TGAb (r=−0.045; p=0.794) (Table 2). These results suggested that resistin levels might associate with thyroid function but not autoimmune antibodies.

**Table 1:** Clinical characteristics of GD patients and healthy controls

	Initial GD (uGD)	Euthyroid GD (eGD)	TRAb-negative GD (nGD)	Healthy controls (hCD)
**N**	39	39	39	25
**Gender**				
** Female**	31	31	31	19
** Male**	8	8	8	6
**BMI**	19.65±2.21	21.40±2.34^a1^	22.19±3.16^b1^	21.32±2.24^c2^
**Thyroid Function Examinations**				
** FT3 (pmol/liter)**	23.60±13.08	4.26±0.75^a1^	4.13±0.50^b1^	4.50±0.44^c1^
** FT4 (pmol/liter)**	33.44±16.12	10.66±2.40^a1^	12.60±1.54^b1^	15.2±1.83^c1^
** s-TSH (mIU/liter)**	0.00097±0.00646	1.69899±3.13408^a1^	2.28984±1.13730^b1^	1.64062±0.64855^c1^
**Thyroid Autoantibodies**				
** TPOAb (IU/ml)**	423.31±383.9	469.07±248.65^a2^	139.11±138.46^b2^	0.28±0.25^c1^
** TRAb (IU/liter)**	8.65±9.86	3.60±7.42^a1^	0.74±0.37^b1^	0.77±0.37^c1^
** TGAb (IU/ml)**	304.54±315.20	246.46±398.89^a2^	74.75±110.43^b2^	3.49±3.29^c1^

uGD compared with eGD: ^a1^ P<0.05,^a2^ P>0.05;

uGD compared with nGD: ^b1^ P<0.05,^b2^ P>0.05;

uGD compared with hCD: ^c1^ P<0.05,^c2^ P>0.05.

**Table 2:** Pearson's correlation analysis of resistin associated with classic GD diagnostic parameters

GD Parameters	r	p
**FT3**	−0.375	0.022
**FT4**	−0.352	0.028
**s-TSH**	−0.156	0.342
**TRAb**	−0.199	0.224
**TPOAb**	0.57	0.735
**TGAb**	−0.045	0.794

### Correlation of serum resistin level with neutrophile counts in GD patients

**Figure 2:**
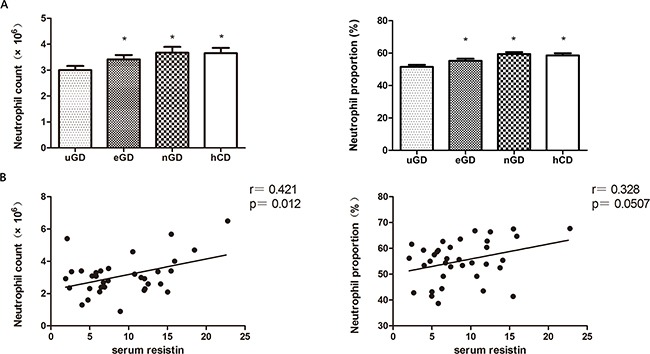
The correlation of serum resistin with neutrophil counts **A.** Circulating neutrophils counts and proportion in uGD, eGD, nGD and hCD. **B.** The serum resistin level in uGD patients is positive correlated with circulating neutrohpils. * P<0.05.

As human resistin was considered to be secreted from circulating leukocytes, we then analyzed the correlation of resistin with each components of leukocytes. We measured the leukocytes count and proportion in GD patients and analyzed their changes during GD treatments (Supplementary Table S1). The results showed that both neutrophile proportion (Neu%) (51.18±7.19 vs 58.34±6.84; p=0.02) and neutrophile count (NEUT) (2.95±0.97×10^6^ vs 3.75±1.04×10^6^; p=0.007) were declined in uGD patients and recovered in eGD (Neu%: 55.63±7.72; NEUT: 3.50±1.05×10^6^) and nGD (Neu%: 59.04±5.83; NEUT: 3.74±1.48×10^6^) (Figure 2A). Pearson's correlation analysis showed that the serum resistin level was positively correlated with absolute neutrophile count (r=0.421, p=0.012) and proportion (r=0.328, p=0.0507) in uGD patients (Figure 2B), which indicating a connection between resistin and neutrophils.

### Decreased expression of resistin in neutrophils after long-time T3 treatment

**Figure 3:**
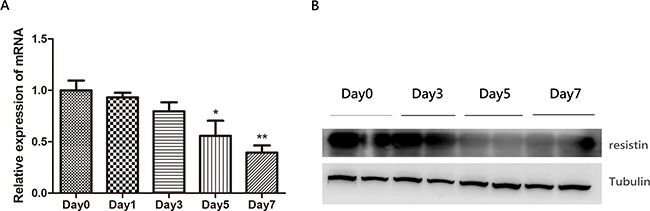
The effect of thyroid hormone T3 (100ng/ml) on resistin expression in neutrophil cell line NB4 The mRNA **A.** and protein **B.** expression of resistin in NB4 is suppressed upon T3 treatment in a time-dependent manner.*: compared with Day0. * P<0.05* * P<0.01.

In order to explore the mechanism of resistin decrease in GD patients, we treated the neutrophil cell line NB4 with T3 at different time points. As illustrated in Figure 3, the resistin mRNA expression in NB4 was unchanged during the first 3 days but declined from the fifth day. Western bolt showed the same result as of mRNA, indicating the long-time T3 treatment on NB4 cell line could repress resistin expression.

## DISCUSSION

Graves' disease is an autoimmune disease of the thyroid gland in which autoantibodies bind to the thyrotropin receptor on thyroid follicular cells, thus over stimulating the function of thyroid gland leading to excess production of thyroid hormones, which have a big punch to metabolism status. [1, 5, 6]. Despite autoantibodies, altered actions and levels of several cytokine and adipokines have been reported in GD patients, including resistin. It has been reported that drug-induced hypothyroid rats exhibit elevated levels of adipose tissue resistin mRNA, which become almost undetectable after inducing thyrotoxicosis with exogenous T4 administration [29]. In humans, there also have been some investigations exploring the relationship of serum resistin and thyroid function, especially in untreated GD patients. However, the studies so far have yielded controversial results (Table 3).

**Table 3:** Studies showing the serum resistin level in hyperthyroid patients and its correlation with clinical parameters

Author, year [Ref.]	Patients	Thyrotoxicosis	Treated thyrotoxicosis	T3 and T4	TSH	BMI	TRAb
**Pedro Iglesias, 2003^a^**	n = 20	↓	↔ (↓^d^)				
**Yaturu, 2004^b^**	n = 69	↑		+	−		
**Krassas, 2005^a^**	n = 43	↑	↓	○	○	○	
**L. Sieminska 2008^a^**	n = 76	↔		○	○		○
**Bossowski A 2010^c^**	n = 31	↓					
**El Gawad SS, 2012^a^**	n =40	↑	↓	○	○		
**Ceren Eke Koyuncu, 2013^a^**	n = 30	↑			+		

a Compared with euthyroid control.

b Compared with hypothyroidism patients.

c Compared with simple goiter and Hashimoto's thyroiditis

d After adjusting for BMI.

Arrows represent changes in resistin levels: ↓ decreased, ↑ elevated, and ↔ unchanged. Correlation with clinical parameters: + positive correlation, − negative correlation, ○ no correlation

The results of our study showed that serum resistin level was significantly decreased in untreated GD patients compared with healthy controls, which is in accordance with Iglesias and Bossowski A's study [24, 25]. But conflict with Iglesias study which exhibited resistin level had no change after treatment, our study found the resistin level increased to normal range when the patients returned to a euthyroid state. The main drawback of that study was the small number of patients enrolled and their patients received different treatments. Despite MMI treatment, four patients had an additional therapy with radioactive iodine (^131^I) and one was treated by subtotal thyroidectomy, both of which might have an effect on metabolism status severely. Study from Bossowski only found a decrease of resum resistin in untreated GD patients compared with hypothyroidism, but they did not explore the change of resistin level after normalization of thyroid function. There also have been several investigations demonstrating an upregulation of serum resistin concentration in hyperthyroid patients. But in Yaturn study, though the patients showed homogenous uptake of ^131^I radioiodine, the TRAb level of GD patients were not shown which was essential in determining Graves disease [30]. While in our study, all patients possessed abnormally high level of TRAb and exhibited much higher concentrations of FT3 and FT4. In addition, they did not undertake a follow-up survey. What's more, patients in Krassas, El Gawad and Ceren Eke studies were not definitely refereed to Graves disease which was different with our enrollment criteria [26, 27, 31]. Besides, though they detected the serum resistin level before and after normalization of thyroid function, all of them were not case-control studies while in our study, we compared the same 39 GD patients in different therapy stages to illustrate the variation trends in eGD and nGD. There was also a paper finding no change between GD patients and healthy controls [28]. This contradiction may be as a result of the components of GD patients consisted of 26 subjects without ophthalmopathy and 50 subjects with ophthalmopathy, which was different from our study including only GD patients without ophthalmopathy.

In our study, the reduction of serum resistin level in uGD patients remained statistically significant even rule out the effect of BMI, indicating the decrease of serum resistin was independent of BMI loss. And it is in accordance with other studies demonstrating human resistin level was not simply associated with fat tissue but the inflammation degree occured in the whole body [11]. Besides, the serum resistin levels in uGD patients held a negative correlation with FT3 and FT4, but not with autoimmune antibodies. Coupled with the fact that resistin was increased after normalization of thyroid hormones, it can be concluded that the change of serum resistin was associated with thyroid function but not the autoimmune processes involved in GD development.

Human resistin, produced and secreted from immune cells including monocyte, lymphocyte and neutrophil, has been recognized to plays an essential role in inflammatory and autoimmune disorders [10]. What's more, a previous study reported that the number of blood leucocyte was an independent explanatory factor for circulating resistin concentration [32]. And it is reported that the highest resistin protein level was detected in circulating neutrophils and it was stored in neutrophil granules being released upon challenge with inflammatory stimuli [33, 34]. In our study, the neutrophils counts and neutrophils proportion were downregulated in our uGD patients, which is consistent with several previous studies [35, 36]. And after effective treatments, NEUT and Neu% gradually increased to normal range. The Pearson's test showed there was a significantly positive correlation between resistin level with NEUT in uGD patients. To testify whether the high level of circulating thyroid hormones could supress the expression of resistin in neutrophils, we stimulated NB4, a neutrophil cell line, with T3 to ultimately found both of the mRNA and protein level of resistin were declined in a time-dependent manner, further banking up our viewpoint above.

Several studies have revealed that excessive reactive oxygen species (ROSs) were produced in GD patients [37, 38, 39]. And when antioxidant supplementation was added to MMI, euthyroidism was more rapidly achieved [40], inferring the plethoric ROSs might be a contributor of GD development. In addition, neutrophils produce higher levels of oxidative species in GD patients than controls [41]. However, it has been reported that resistin decreased oxidative burst in neutrophils [42, 43]. From our data, we demonstrated that supplemented with T3, the neutrophils could produced less amount of resistin, so that the suppressive effect of resistin on ROSs production might be crippled, resulting in a robust synthesis of detrimental ROSs after compensating for the reduction of neutrophils.

In conclusion, we reported a down-regulation of serum resistin in untreated GD patients and its downregulation was irrelevant with weight loss. The serum resistin was mainly secreted from neutrophils and was significantly downregulated by thyroid hormones. Since previous reports indicated that resistin could suppress ROSs synthesis, we speculated that the reduction of serum resistin in uGD patients might increase ROS production and aggravate the immune disorders, which deserved further exploration.

## MATERIALS AND METHODS

### Patients

A total of 39 Graves' disease patients (8 males and 31 females) who met criteria for untreated GD (uGD) and 25 healthy control doners (hCD) were enrolled in our study from the outpatient Department of Rui-jin Hospital affiliated to Shanghai Jiao Tong University. GD patients were diagnosed with the following strateges: 1) untreated GD: newly diagnosed without any previous treatment; the precence of representative hyperthyroidism symptoms; clinical evaluation: physical examination, thyroid ultrasonography; laboratory examinations: serum concentrations of free T3 (FT3), free T4 (FT4), sensitive TSH (s-TSH), thyroperoxidase antibody (TPOAb), all of which were measured by automated chemiluminescent immunoassays (Architecti2000SR; AbbottLaboratories, Chicago, IL). Serum levels of thyrotrophin receptor antibody (TRAb) and thyroglobulin antibodies (TGAb) were measured by radioreceptor assays with commercial kits (DiaSorin, Stillwater, MN); 2) euthyroid GD: uGD with 3-4months methimazole (MMI) treatments, consequently obtained normal levels of s-TSH, FT3 and FT4 but TRAb level was just slightly decreased compared with uGD stage; 3) TRAb negative-conversion: uGD patients treated with MMI for 1-3 years whose s-TSH, FT3, FT4 as well as TRAb returned to normal range and maintained stable for at least 3 months. 25 age and sex-matched healthy volunteers without any history of thyroid dysfunction past or present were included in this study. Leukocyte counts were analysed by Sysmex XN-9000 (Sysmex Corporation, Kobe, Japan) blood cell analyser by flow cytometry using semiconductor laser. All analyzers were calibrated and maintained according to the manufacturer's instructions. Our study has been approved by the ethics reviews committee from Shanghai Jiao Tong University School of Medicine.

### Detection of serum resistin by ELISA

5ml fresh whole blood was obtained and then centrifuged at 3000 rpm for 5 min. The serum was collected immediately and then stored properly (−80°C) until following ELISA measurement. The resistin concentrations of serum were tested in duplicates by ELISA kit (R&D systems, Minnepolis, MN) according to the manufacture's instructions.

### Cell cultures and treatment

Human neutrophile cell line NB4 were cultured in RPMI 1640 supplemented with 100 U/mL penicillin, 0.1 mg/mL streptomycin, and 10% deactivated FBS maintained in 5% CO2 at 37°C. Cells were treated with or without 100ng/ml T3 for indicated time.

### RNA extraction and real-time PCR

Total RNA was isolated from treated cells using TRIZOL regent (Invitrogen, Carlsbad, CA) and 1mg RNA was converted into first-strand cDNA with the First Strand cDNA Synthesis Kit (Progema, Msdison, WI) according to the manufacture's instructions. Real-time PCR was performed using SYBR Master Mix (Takara, Shiga, Japan) on an ABI Prism 7900HT (Applied Biosystems, Foster City, CA). PCR array data were calculated using ΔΔCt method and normalized against housekeeping gene GAPDH. All the primer sequences used are list below (5′->3′):

RT-hGAPDH-F: GGCATGGACTGTGGTCATGAG

RT-hGAPDH-R: TGCACCACCAACTGCTTAGC

RT-hResistin-F: CTGTTGGTGTCTAGCAAGACC

RT-hResistin-R:CTGTTGGTGTCTAGCAAGACC

### Statistical analysis

Descriptive data are shown as means ± SD. *P* values less than 0.05 are considered to be significant. The value of serum resistin level was logarithmically transformed before statistical analysis to approximate normal distributions. Comparisons among serum parameters in different state of thyroid function and healthy donors were performed using *t* test. Correlations between the different variables were analyzed by simple correlation using Pearson's test. All statistical analysis was performed using the SPSS version 13.0. All graphics were performed by GRAPH PAD PRISM 5.0.

## SUPPLEMENTARY TABLE


