# Supplementary material for: Down-regulated resistin level in consequence of decreased neutrophil counts in untreated Grave's disease
Source: Oncotarget. 2016 Sep 14;7(48):78680–7. doi: 10.18632/oncotarget.12019 (PMC5346669; doi:10.18632/oncotarget.12019)
Supplement: Supplementary file 1 [file oncotarget-07-78680-s001.pdf]

Down-regulated resistin level in consequence of decreased neutrophil counts in untreated Grave's disease

SUPPLEMENTARY TABLE

Supplementary Table S1: The circulating leukocyte in uGD, eGD, nGD and healthy controls

Inflammatory cells distributions	uGD	eGD	nGD	hCD
**WBC**	5.84±1.00	6.08±1.65[a2]	6.30±2.73[b2]	6.30±1.46[c2]
**Neutrophil count**	2.95±0.97	3.50±1.05[a1]	3.74±1.48[b1]	3.75±1.04[c1]
**Neutrophil%**	51.18±7.19	55.63±7.72[a1]	59.04±5.83[b1]	58.34±6.84[c1]
**Lymphocyte count**	1.77±0.73	2.07±0.55[a2]	1.99±0.82[b2]	2.00±0.54[c2]
**Lymphocyte%**	31.84±9.60	34.35±5.57[a2]	32.73±9.03[b2]	32.51±6.30[c2]
**Monocyte count**	0.50±0.15	0.39±0.15[a1]	0.40±0.22[b1]	0.39±0.12[c1]
**Monocyte%**	8.73±2.86	6.50±2.17[a1]	6.35±1.61[b1]	4.19±3.03[c1]

uGD compared with eGD: [a1] $P<0.05$, [a2] $P>0.05$;
uGD compared with nGD: [b1] $P<0.05$, [b2] $P>0.05$;
uGD compared with hCD: [c1] $P<0.05$, [c2] $P>0.05$;
